# SHMT1 inhibits the metastasis of HCC by repressing NOX1-mediated ROS production

**DOI:** 10.1186/s13046-019-1067-5

**Published:** 2019-02-12

**Authors:** Changwei Dou, Qiuran Xu, Jie Liu, Yufeng Wang, Zhenyu Zhou, Weifeng Yao, Kai Jiang, Jian Cheng, Chengwu Zhang, Kangsheng Tu

**Affiliations:** 1 Department of Hepatopancreatobiliary Surgery & Minimally Invasive Surgery, Zhejiang Provincial People’s Hospital (People’s Hospital of Hangzhou Medical College), Hangzhou, 310014 Zhejiang Province China; 2 Key Laboratory of Tumor Molecular Diagnosis and Individualized Medicine of Zhejiang Province, Zhejiang Provincial People’s Hospital (People’s Hospital of Hangzhou Medical College), Hangzhou, 310014 Zhejiang Province China; 3 Department of Hepatobiliary Surgery, the First Affiliated Hospital of Xi’an Jiaotong University, Xi’an, 710061 Shaanxi Province China; 4 Department of Hepatobiliary Surgery, Sun Yat-Sen Memorial Hospital, Sun Yat-Sen University, Guangzhou, 510120 Guangdong Province China

**Keywords:** SHMT1, Hepatocellular carcinoma, Metastasis, NOX1, Reactive oxygen species

## Abstract

**Background:**

Hepatocellular carcinoma (HCC) is the most major type of primary hepatic cancer. Serine hydroxymethyltransferase 1 (SHMT1) is recently found to play critical roles in human cancers including lung cancer, ovarian cancer and intestinal cancer. However, the expression, function and the underlying mechanisms of SHMT1 in HCC remain uncovered.

**Methods:**

qRT-PCR, immunohistochemistry and immunoblotting were performed to detect the expression of SHMT1 in HCC tissues and cell lines. HCC cell migration and invasion were determined by Boyden chamber and Transwell assay in vitro, and tumor metastasis was assessed via lung metastasis model in mice. The expression of key factors involved in epithelial-to-mesenchymal transition (EMT) process was evaluated by western blotting.

**Results:**

In this study, data mining of public databases and analysis of clinical specimens demonstrated that SHMT1 expression was decreased in HCC. Reduced SHMT1 level was correlated with unfavorable clinicopathological features and poor prognosis of HCC patients. Gain- and loss-of-function experiments showed that SHMT1 overexpression inhibited the migration and invasion of HCCLM3 cells while SHMT1 knockdown enhanced the metastatic ability of Hep3B cells. Furthermore, qRT-PCR and western blotting showed that SHMT1 inhibited EMT and matrix metallopeptidase 2 (MMP2) expression. In vivo experiments showed that SHMT1 suppressed the lung metastasis of HCC cells in mice. Mechanistically, SHMT1 knockdown enhanced reactive oxygen species (ROS) production, and thus promoted the motility, EMT and MMP2 expression in Hep3B cells. Furthermore, NADPH oxidase 1 (NOX1) was identified to be the downstream target of SHMT1 in HCC. NOX1 expression was negatively correlated with SHMT1 expression in HCC. Rescue experiments revealed that NOX1 mediated the functional influence of SHMT1 on HCC cells.

**Conclusions:**

These data indicate that SHMT1 inhibits the metastasis of HCC by repressing NOX1 mediated ROS production.

**Electronic supplementary material:**

The online version of this article (10.1186/s13046-019-1067-5) contains supplementary material, which is available to authorized users.

## Background

Hepatocellular carcinoma (HCC), one of most common malignancies, is the third frequent cause of cancer-related mortality, with over 600,000 newly diagnosed cases annually [1]. Curative treatment for HCC including surgical resection and liver transplantation are only available for patients in early stage [2–4]. For HCC patients in advanced stages, the overall prognosis remains poor due to lack of effective treatments [5]. The occurrence of intrahepatic or systemic metastasis is an important reason for the unsatisfactory prognosis of HCC patients in advanced stage. However, the molecular mechanism underlying the metastasis of HCC remains largely unknown.

Serine hydroxymethyltransferases (SHMTs) is the key protein regulating one carbon (methyl) metabolism and comprises the cytoplasmic isozyme (SHMT1) and mitochondrial isozyme (SHMT2) [6]. SHMT1 catalyzes the conversion of serine and tetrahydrofolate to glycine and 5, 10 methylene tetrahydrofolates. This reversible conversion provides one-carbon units for nucleotide synthesis, and affects DNA methylation status and NADH/NADPH production [7, 8]. In recent years, SHMT1 was found to be a novel cancer-associated protein in human cancers [9–19]. In lung cancer [9, 10], ovarian cancer [11] and breast cancer [12], SHMT1 was found to act as onco-protein and promote the progression of these cancers. However, the expression and function of SHMT1 in HCC remain uncovered.

In this study, both data mining of public databases and confirmatory experiments in clinical specimens revealed that SHMT1 expression was significantly decreased in HCC. Decreased SHMT1 expression was correlated with adverse clinical features and poor prognosis of HCC patients. Functionally, we found that SHMT1 suppressed the metastasis, epithelial–mesenchymal transition (EMT) and matrix metalloproteinase-2 (MMP2) expression of HCC cells using both gain- and loss- of function assays. Mechanically, this study demonstrated that SNMT1 inhibited reactive oxygen species (ROS) production, and thus decreased the metastatic ability of Hep3B cells. Moreover, we elucidated that NADPH oxidase 1 (NOX1), whose expression was under the control of SHMT1, was responsible for the inhibitory effect of SHMT1 on ROS production and metastasis of HCC cells.

## Methods

### Clinical tissues

Clinical specimens including HCC tissues and adjacent non-tumor tissues were collected from 120 HCC patients who received surgical resection in the Department of Hepatobiliary Surgery, Sun Yat-Sen Memorial Hospital. The diagnosis of HCC was confirmed by pathologists. All patients did not receive any chemotherapy or interventional treatment before surgical resection. Freshly-resected HCC tissues were stored at − 80 °C for qRT-PCR assay, and formalin-fixed and embedded in paraffin for immunohistochemistry (IHC) staining. The domestic information and clinical characteristics of HCC patients are demonstrated in Table 1.

**Table 1:** Correlation analysis between the clinical features and SHMT1 expression in HCC

Characteristics of patients		No. of patients	SHMT1 level		*P*
			Low	High	
Age (y)	< 60	86	47	39	0.149
	≥60	34	24	10	
Sex	Male	103	60	43	0.187
	Female	17	13	4	
HBV infection	Absent	15	10	5	0.587
	Present	105	61	44	
Cirrhosis	Absent	23	18	5	0.058
	Present	97	53	44	
AFP level	< 20	36	17	19	0.105
	≥20	84	54	30	
Tumor size (cm)	< 5	44	22	22	0.129
	≥5	76	49	27	
Vascular invasion	Absent	56	26	30	0.009*
	Present	64	45	19	
Edmondson-Steiner grading	I + II	76	39	37	0.021*
	III + IV	44	32	12	
TNM tumor stage	I + II	39	17	22	0.019*
	III + IV	81	54	27	

HCC, hepatocellular carcinoma; HBV, hepatitis B virus; AFP, alpha fetoprotein; TNM, tumor-node-metastasis. ^*^ Statistically significant

### Cell culture

Four HCC cell lines (HCCLM3, SMMC7721, Huh7, and Hep3B), and the human immortalized normal hepatocyte cell line (LO2) were purchased from the Cell Bank of the Chinese Academy of Sciences (Shanghai, China). All cells were cultured in Dulbecco’s modified Eagle medium (DMEM, Gibco, Grand Island, NY, USA) supplemented with 10% fetal bovine serum (FBS, Gibco) along with 100 units/mL penicillin and 100 μg/mL streptomycin (Sigma, St-Louis, MO, USA) and maintained in a humidified air containing 5% CO_2_ at 37 °C.

### Reverse transcription-quantitative polymerase chain reaction (RT-qPCR)

PCR amplifications for the quantification of SHMT1, NOX1, NOX2, NOX3, NOX4, NOX5, DUOX1, DUOX2 and GAPDH were performed using a SYBR® Premix Ex Taq™ II (Perfect Real-Time) kit (Takara Bio, Otsu, Japan) and an ABI PRISM 7300 Sequence Detection system (Applied Biosystems, Foster City, CA, USA). GAPDH was used as internal control. The primers used for qRT-PCR were listed as below: NOX1 forward primer: 5’-GTTTTACCGCTCCCAGCAGAA-3′, reverse primer: 5’-GGATGCCATTCCAGGAGAGAG-3′; NOX2 forward primer: 5’-GGAGGATCCGTGGTCACTCACCCTTTCAA-3′, reverse primer: 5’-CCACTCGAGCTCATGGAAGAGACAAGTTAG-3′; NOX3 forward primer: 5’-GCAGGATCCGTGGTAAGCCACCCCTCTG-3′, reverse primer: 5’-GCTGAATTCAGAAGCTCTCCTTGTTGTAAT-3′; NOX4 forward primer: 5’-GCAGGATCCGTCATAAGTCATCCCTCAGA-3′, reverse primer: 5’-GCTGTTAACGTCGACTCAGCTGAAAGACTCTTTAT-3′; NOX4 forward primer: 5’-GCAGGATCCACTATCTGGCTGCACATTCG-3′, reverse primer: 5’-GCTGAATTCCTAGAAATTCTCTTGGAAAAATC-3′; NOX5 forward primer: 5′- GGAGGATGCCAGGTGGCTCCGGT-3′, reverse primer: 5’-AGCCCCACTACCACGTAGCCC-3′; DUOX1 forward primer: 5’-TTCACGCAGCTCTGTGTCAA-3′, reverse primer: 5’-AGGGACAGATCATATCCTGGCT-3′; DUOX2 forward primer: 5’-ACGCAGCTCTGTGTCAAAGGT-3′, reverse primer: 5’-TGATGAACGAGACTCGACAGC-3′; E-cadherin forward primer: 5’-TCCCATCAGCTGCCCAGAAA-3′, reverse primer: 5’-TGACTCCTGTGTTCCTGTTA-3′; N-cadherin forward primer: 5’-TTTGATGGAGGTCTCCTAACACC-3′, reverse primer: 5’-ACGTTTAA CACGTTGGAAATGTG-3′; vimentin forward primer: 5’-GACGCCATCAACACCGAGTT-3′, reverse primer: 5’-CTTTGTCGTTGGTTAGCTGGT-3′; MMP2 forward primer: 5′- GACAACGCCCCCATACCAG-3′, reverse primer: 5’-CACTCGCCCCGTGTGTTAGT-3′; Snail1 forward primer: 5’-GCTGCAGGACTCTAATCCAGA-3′, reverse primer: 5’-ATCTCCGGAGGTGGGATG-3′; Twist1 forward primer: 5’-GGCCGGAGACCTAGATG-3′, reverse primer: 5′- ACGGGCCTGTCTCGCTTTCT-3′; Zeb1 forward primer: 5’-GCCAACAGACCAGACAGTGTT-3′, reverse primer: 5′- TTTCTTGCCCTTCCTTTCTG-3′; GAPDH forward primer: 5’-AGGGCTGCTTTTAACTCTGGT-3′, reverse primer: 5’-CCCCACTTGATTTTGGAGGGA-3′.

### Western blot

Western blots were performed as we previously described [20, 21]. The following antibodies were used in this study including SHMT1 (Abcam, ab186130), E-cadherin (Cell signaling technology, #14472), N-cadherin (Cell signaling technology, #13116), Vimentin (Cell signaling technology, #5741), MMP2 (Cell signaling technology, #40994), NOX1 (Abcam, ab55831), Snail (Cell signaling technology, #3879), Twist1 (Abcam, ab50887), Zeb1 (Abcam, ab245282) and GAPDH (Abcam, ab9485).

### Immunohistochemical staining (IHC)

Immunohistochemistry was performed on paraformaldehyde-fixed paraffin sections. The detailed staining procedure was conducted as described previously [20, 22]. The slides were incubated with SHMT1 antibody (1:50), NOX1 (1:100), and AFP (1:400).

### Boyden chamber and Transwell assay

Boyden chamber assay (NeuroProbe, Gaithersburg, MD, USA) was used to evaluate the migration ability of HCC cell as previously described [20, 23]. Transwell assays were performed in 24 well plates with Transwell inserts equipped with 8-μm pores (Nalge Nunc International Corp, Naperville, IL, USA) coated with Matrigel at 1:8 dilution (Becton Dickinson Labware, Bedford, MA, USA) as previously described [20].

### Lentiviral or retroviral transduction and cell transfection

Lentiviral SHMT1 shRNA plasmid (RHS4430) and lentiviral negative control shRNA plasmid (RHS4346) were obtained from Open Biosystems. SHMT1 cDNA (A4840, Genecopia) was cloned into pMMP vector (Addgene). For retrovirus or lentivirus package, constructs and packaging plasmids were transfected into 293 T cells using Effectene (Qiagen, Hilden, Germany) as we previously described [24]. The media containing the retrovirus or lentivirus were collected 72 h after transfection, and purified virus particles were used to infect HCC cell lines. NOX1 siRNA (AM16708) and negative control siRNA (AM4611) were obtained from Thermo Fisher Scientific. NOX1 cDNA (H1873, Genecopia) was cloned into pcDNA3.1 vector (Invitrogen, Carlsbad, CA) as previously described, to construct the SHMT1-pcDNA3.1 vector. NOX1 expressing vector or NOX1 siRNA was transfected into HCC cells with Lipofectamine 200 following manufacture’s protocol. qRT-PCR and western blot were performed to confirm the efficacy of viral transduction and cell transfection.

### Measurement of cellular ROS and mitochondria membrane potential (MMP)

The ROS level in HCC cells was evaluated using the ROS assay kit (GMS10016.2, Gemmed Scientifics, USA) following manufacture’s protocol. HCC cells were incubated with DCFH-DA probe at 37 °C for 30 min, followed by serum-free IMDM medium washing for three times. DCFH-DA fluorescence was measured by a Micro Fluorescence Reader with the excitation of 490 nm. Mitochondria-derived ROS was measured by staining HCC cells with MitoSox Red (Life Technologies, US) and reading the absorbance with the excitation of 510/580 nm based on manufacturer’s instructions. To evaluate mitochondrial membrane potential (MMP), cells were stained with 5,50,6,60-tetrachloro-1,10,3,30-tetraethyl benzimidazolylcarbocyanine iodide (JC-1, Invitrogen) and then were analyzed on a Beckman Coulter FC500.

### In vivo metastasis experiment

BALB/c nude mice were used for tail vein injection experiments, to evaluate the metastatic ability of HCC cells in vivo. Hep3B cells with SHMT1 knockdown (1 × 10^6^) or negative control Hep3B cells (1 × 10^6^), and HCCLM3 cells overexpressing SHMT1 or control HCCLM3 cells were injected into the tail veins of nude mice. 8 weeks after tail vein injection, the lungs of nude mice were removed and embedded in paraffin for H&E staining and immunohistochemistry staining of AFP (Cell signaling technology, #4448). Animal protocols were approved by the Institutional Animal Care and Use Committee of Xi’an Jiaotong University.

### Statistical analysis

Results are presented as Mean ± SEM. Significance between groups was evaluated with the SPSS statistical package (SPSS, Chicago, IL, USA) and GraphPad Prism 5 software (San Diego, CA, USA), using Pearson chi-squared test, two-tailed Student’s t test, Kaplan–Meier plot, Spearman’s rank correlation coefficient or ANOVA analysis when appropriate. *P* < 0.05 was considered to be statistical difference between groups.

## Results

### SHMT1 expression is decreased in HCC tissues and cells

**Fig. 1:**
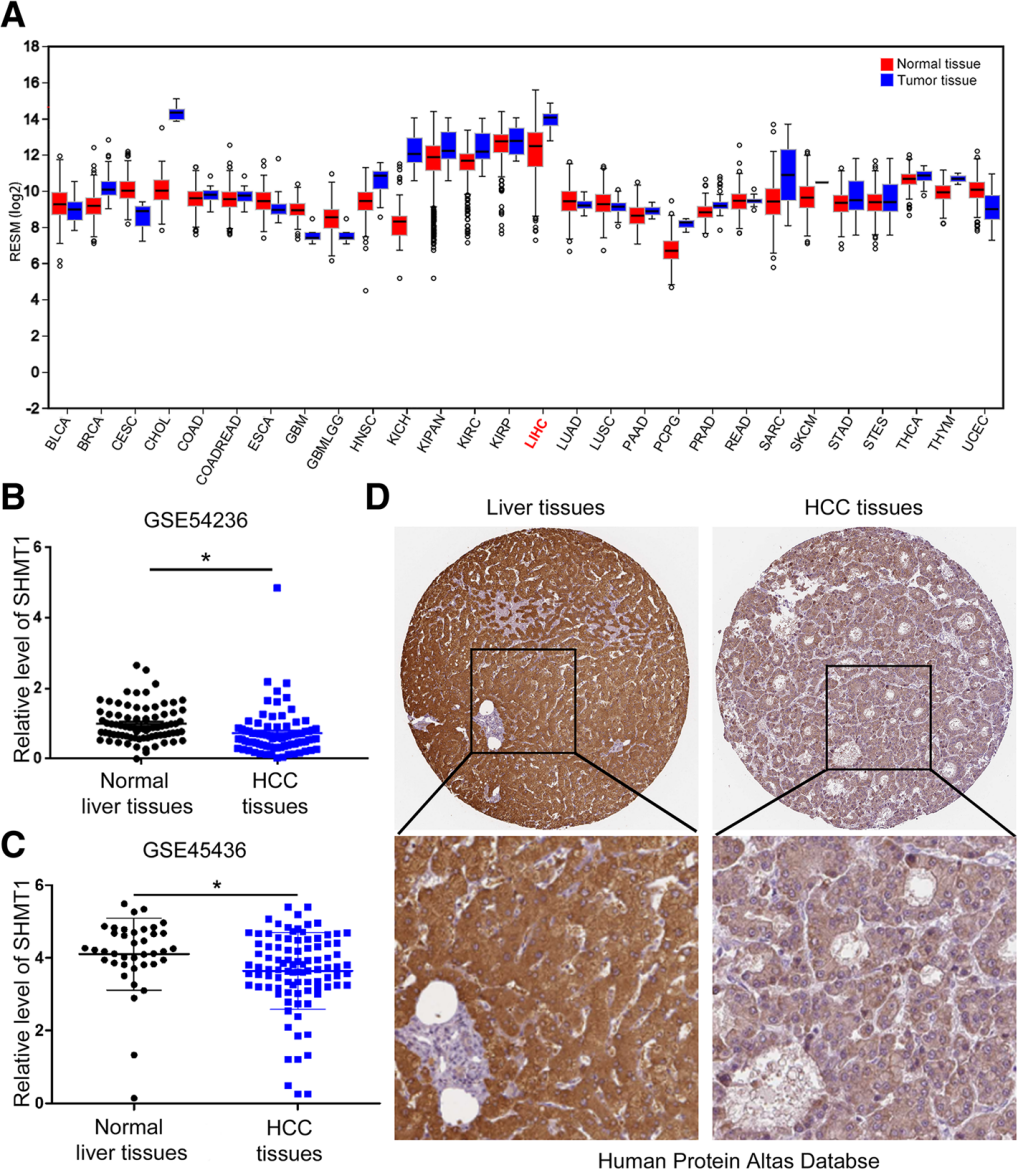
SHMT1 expression data extracted from HCC-related database. (**a**) Differential expression analysis of TUFT1 in 37 types of human cancers on the Firebrowse website. SHMT1 level showed Tumor tissues were labeled as red while the corresponding normal tissues are labeled in blue. (**b**) and (**c**) The expression level of SHMT1 mRNA in GSE54236 database and GSE6764 database. (**d**) Representative IHC staining of SHMT1 protein in Human Protein Atlas website (https://www.proteinatlas.org/). *P < 0.05

To evaluate the expression of SHMT1 in HCC, we first explored the publicly available database compiled at the FireBrowse website (www.firebrowse.org). Among 28 human cancers, 11 cases showed elevated TUFT1 levels, while 17 cases showed decreased SHMT1 levels, compared with the corresponding normal tissues (Fig. 1A). Among these human cancers, HCC (LIHC in Figure1A and Additional file 1: Figure S1A) tissues showed a substantial decrease in SHMT1 levels compared with normal liver. To validate the data obtained from FireBrowse website, we employed the “R2: Genomics Analysis and Visualization Platform” (http://r2.amc.nl) to explore SHMT1 expression in Gene Expression Omnibus (GEO) datasets of HCC. As shown in Fig. 1B and C, 2 GEO datasets (GSE54236 and GSE45436) consistently showed decreased SHMT1 mRNA level in HCC specimens compared with adjacent non-tumor liver specimens (*P* < 0.05). Moreover, IHC staining data from the Human Protein Atlas website (https://www.proteinatlas.org/) showed that SHMT1 protein in HCC tissues had significantly decreased staining intensity compared with that in normal liver tissues (Fig. 1D).

**Fig. 2:**
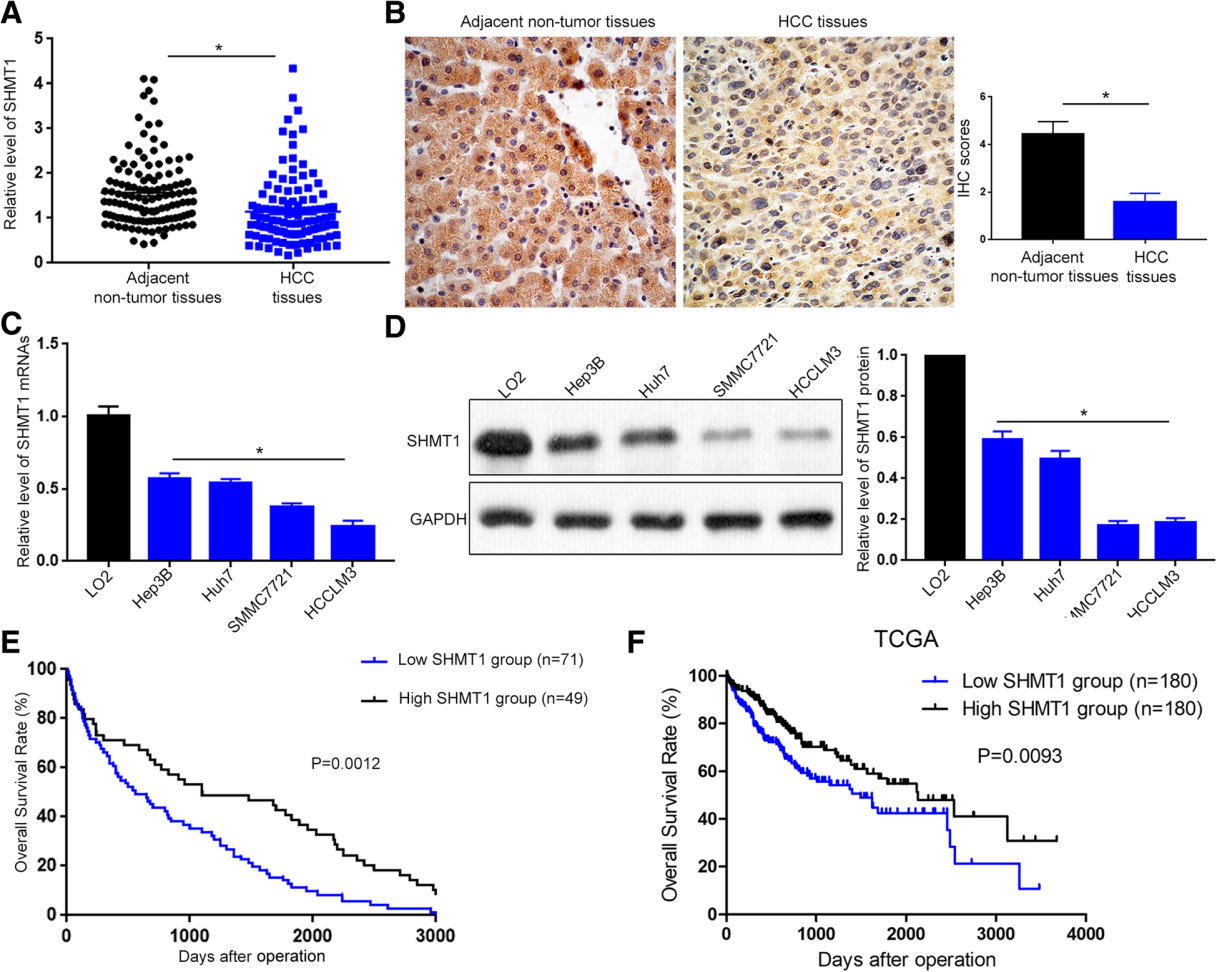
SHMT1 expression is decreased in HCC and predicts poor prognosis of HCC patients. (**a**) qRT-PCR was performed to evaluate SHMT1 mRNA level in 120 pairs of HCC tissues and adjacent non-tumor tissues. (**b**) IHC staining was performed to evaluate SHMT1 protein level in 40 pairs of HCC tissues and adjacent non-tumor tissues. (**c**) qRT-PCR was performed to measure SHMT1 mRNA level in HCC cell lines and human immortalized hepatocyte LO2 cells. (**d**) Western blot was performed to measure SHMT1 mRNA level in HCC cell lines and human immortalized hepatocyte LO2 cells. (**e**) Kaplan-Meier analysis was performed for our HCC patients’ cohort to evaluate the association between SHMT1 level and patients’ overall survival. (**f**) Kaplan-meier analysis was performed for HCC patients’ cohort in TCGA database to evaluate the association between SHMT1 level and patients’ overall survival. *P < 0.05

To further validate the data from online datasets, we measured SHMT1 mRNA levels in 120 pairs of HCC tissues and adjacent non-tumor liver specimens. qRT-PCR showed that SHMT1 mRNA level was significantly decreased in HCC tissues (Fig. 2A, P < 0.05). IHC staining confirmed that HCC tissues had significant decreased staining intensity of SHMT1 protein compared to noncancerous tissues (Fig. 2B, *P* < 0.05). Furthermore, qRT-PCR and western blot were performed to measure the mRNA and protein level of SHMT1 in HCC cell lines and LO2 cell. Compared with the immortalized hepatocyte LO2, four HCC cell lines showed significantly decreased level of SHMT1 (Fig. 2C and D). Altogether, these data confirm that SHMT1 expression level is significantly decreased in HCC.

### Clinical significance of reduced SHMT1 expression in HCC specimens

To investigated the clinical significance of SHMT1 in HCC, we divided HCC patients into low SHMT1 group (*n* = 71) and high SHMT1 group (*n* = 49) with the cutoff value defined as the mean level of SHMT1 mRNA. As shown in Table 1, decreased SHMT1 level was associated with venous infiltration (*P* = 0.009), high tumor grade (*P* = 0.021) and advanced TNM stage (*P* = 0.019). We further divided the HCC cohort based on IHC staining of SHMT1 protein on HCC specimens and found that negative staining of SHMT1 in HCC tissues was correlated with higher AFP level (*P* = 0.016, Additional file 2: Table S1), vascular invasion (*P* = 0.002, Additional file 2: Table S1) and advanced TNM stage (*P* = 0.006, Additional file 2: Table S1). TCGA data from “R2: Genomics Analysis and Visualization Platform” (http://r2.amc.nl) indicated that the expression of SHMT1 was reduced in HCCs with vascular invasion compared to HCCs without vascular invasion (*P* = 0.0441, Additional file 1: Figure S1B). Furthermore, statistical analysis of TCGA data from UALCAN [25] revealed that advanced HCCs had a significant lower expression of SHMT1 compared to early HCCs (*P* = 0.0003, Additional file 1: Figure S1C). Consistently, high-grade HCCs showed an obvious lower expression of SHMT1 as compared with low-grade HCCs (*P* < 0.0001, Additional file 1: Figure S1D). Survival analysis showed that patients with low SHMT1 level had significantly decreased overall survival (Fig. 2E, *P* = 0.0012). Data from TCGA database also validated that decreased SHMT1 level was correlated with poor prognosis of HCC patients (Fig. 2F, *P* = 0.0093). Taken together, these data indicate that SHMT1 plays tumor suppressive role and may function as prognostic marker in HCC.

### SHMT1 inhibits the metastasis, EMT and MMP2 production of HCC cells

**Fig. 3:**
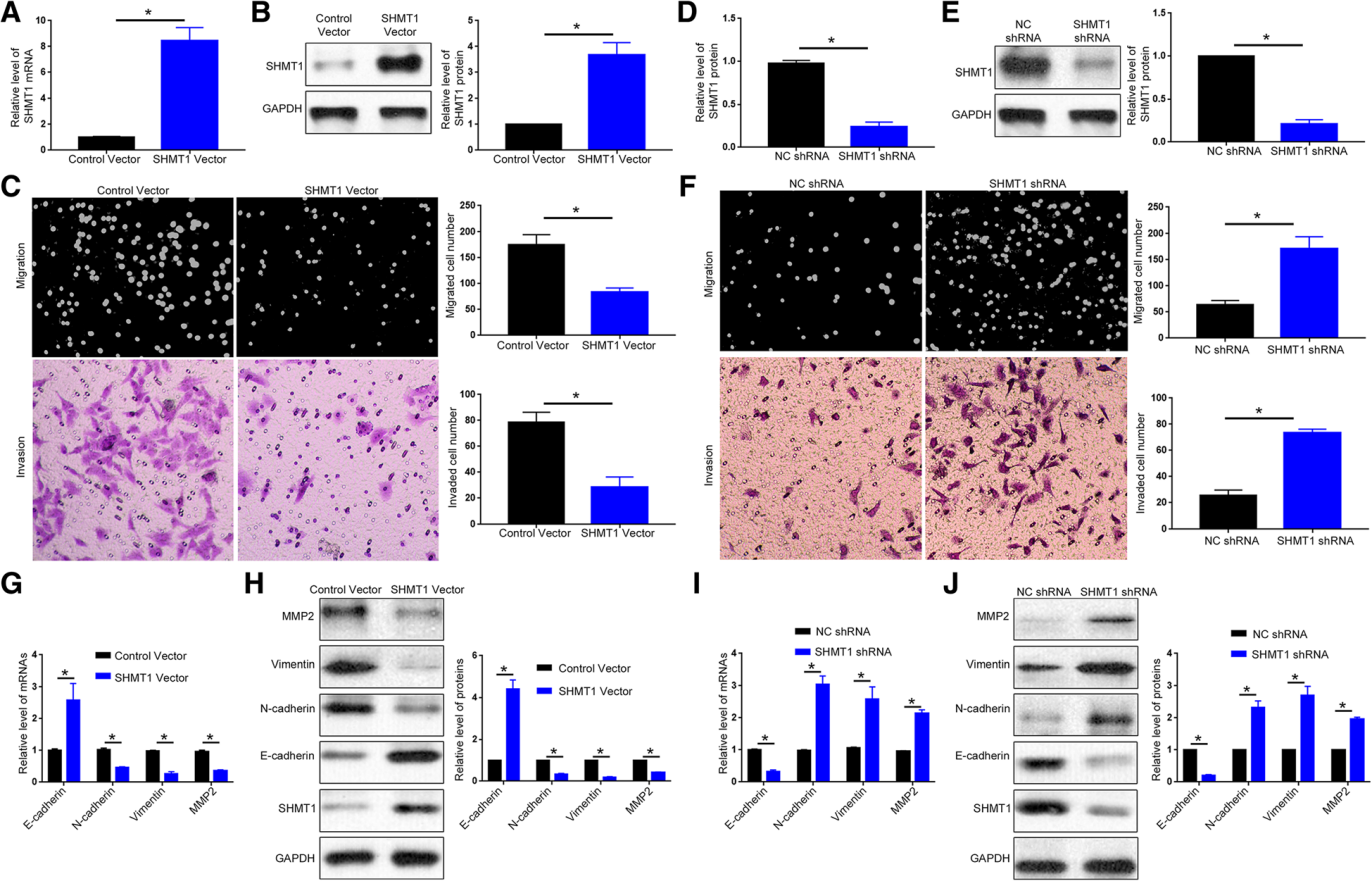
SHMT1 inhibits the migration, invasion, EMT and MMP2 production of HCC cells. Retrovirus encoding empty vector or SHMT1 vector were transduced into HCCLM3 cells. (**a**) qRT-PCR and (**b**) western blot were employed to evaluate the efficacy of retrovirus transduction. (**c**) Boyden chamber and transwell assay were employed to investigate the effect of SHMT1 overexpression on cell migration and invasion. Lentivirus encoding negative control shRNA or SHMT1 shRNA was transduced into Hep3B cells. (**d**) qRT-PCR and (**e**) western blot were employed to evaluate the efficacy of lentivirus transduction. (**f**) Boyden chamber and transwell assay were employed to investigate the effect of SHMT1 knockdown on cell migration and invasion. (**g**) qRT-PCR and (**h**) western blot were employed to evaluate the effect of SHMT1 overexpression on the expression level of E-cadherin, N-cadherin, Vimentin and MMP2. (**i**) qRT-PCR and (**j**) western blot were employed to evaluate the effect of SHMT1 knockdown on the expression level of E-cadherin, N-cadherin, Vimentin and MMP2. *P < 0.05

Since occurrence of metastasis is an important reason for the poor prognosis of HCC patients, we further investigate whether SHMT1 influences the metastasis of HCC cells. Among the four HCC cells, SHMT1 showed highest level in Hep3B cells and lowest level in HCCLM3 cells. Therefore, we performed SHMT1 overexpression in HCCLM3 cells and SHMT1 knockdown in Hep3B cells. Transfection of SHMT1 expressing vector effectively increased SHMT1 mRNA and protein level in HCCLM3 cells (Fig. 3A and B, *P* < 0.05). Boyden chamber assay showed that forced expression of SHMT1 suppressed the migration of HCCLM3 cells (Fig. 3C, *P* < 0.05). Transwell assay demonstrated that SHMT1 overexpression led to significantly decreased invasion of HCCLM3 cells (Fig. 3C, *P* < 0.05). In contrary, transduction of lentivirus with SHMT1 shRNA into Hep3B cells led to significantly decreased level of SHMT1 (Fig. 3D and E, *P* < 0.05), subsequently resulted in enhanced migratory and invasive ability of Hep3B cells (Fig. 3F, *P* < 0.05). To avoid the variability between cell lines, we transfected Hep3B cells with SHMT1 vector and found that forced expression of SHMT1 in Hep3B cells also resulted in significant decrease of cell migration and invasion (*P* < 0.05, Additional file 3: Figure S2). Additionally, we performed MTT assay to investigate whether SHMT1 alteration influenced cell viability. MTT data showed that overexpression or knockdown of SHMT1 did not have any significant influence on cell viability (Additional file 4: Figure S3).

Epithelial-mesenchymal transition (EMT) and MMP production have been widely recognized as critical mechanisms of cancer metastasis [26–28]. Thus, we further explored whether SHMT1 affected EMT and MMP production of HCC cells. As shown in Fig. 3G and H, SHMT1 overexpression in HCCLM3 cells led to significantly increased mRNA and protein level of E-cadherin (*P* < 0.05) and decreased expression of N-cadherin, vimentin and MMP2 (*P* < 0.05). On the other hand, SHMT1 knockdown in Hep3B cells resulted in decreased E-cadherin level and increased expression of N-cadherin, Vimentin and MMP2 (Fig. 3I and J, *P* < 0.05). Previous studies have confirmed that Twist1, Snail1 and Zeb1 were the critical transcriptional factors regulating EMT of cancer cells. We further explored whether SHMT1 affected the expression of these transcriptional factors. qRT-PCR and western blot showed that SHMT1 overexpression inhibited the expression of Twist1 and Snail1 (Additional file 5: Figure S4A) while its knockdown resulted in increased level of these two factors (Additional file 5: Figure S4B). Neither SHMT1 overexpression nor its knockdown had any influence on Zeb1 expression (Additional file 5: Figure S4). In all, these data indicate SHMT1 suppresses the metastatic ability of HCC cells by inhibiting EMT and MMP2 production.

### SHMT1 suppresses the metastasis of HCC cells in vivo

**Fig. 4:**
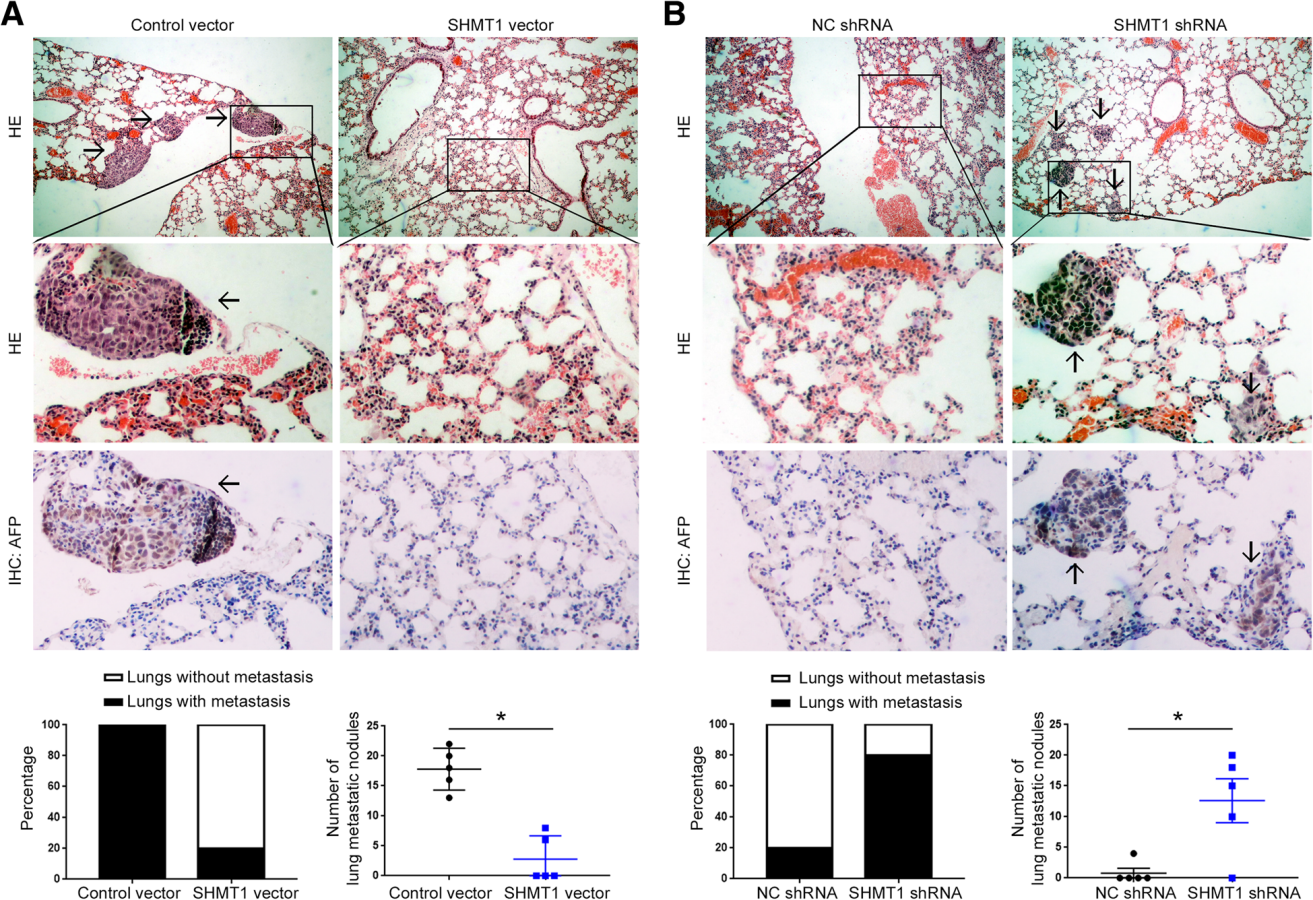
SHMT1 inhibits lung metastasis of HCC cells in nude mice. (**a**) HCCLM3 cells overexpressing SHMT1 or those in control group were injected into nude mice through tail vein, to evaluate the effect of SHMT1 overexpression on in vivo metastasis of HCCLM3 cells. HE staining and IHC staining of AFP were performed to identify the lung metastatic nodules formed by HCCLM3 cells. (**b**) Hep3B cells with or without SHMT1 knockdown were injected into nude mice through tail vein, to evaluate the effect of SHMT1 knockdown on in vivo metastasis of Hep3B cells. HE staining and IHC staining of AFP were performed to identify the lung metastatic nodules formed by Hep3B cells. *P < 0.05

After verifying the functional influence of SHMT1 on cell metastasis in vitro, we further investigated whether SHMT1 affected the metastasis of HCC cells in nude mice. As shown in Fig. 4A, compared with cell in control group, HCCLM3 cells overexpressing SHMT1 showed significantly increased number and rate of lung metastasis in nude mice (*P* < 0.05). Moreover, SHMT1 knockdown significantly enhanced the metastatic ability of Hep3B cells in nude mice, suggested by significantly increased number of metastatic nodules and lung metastasis rate (Fig. 4B, *P* < 0.05). The IHC staining data (Fig. 4A and B), which showed positive AFP staining in these metastatic nodules, further validated these nodules were indeed derived from HCC cells. These data confirm that SHMT1 inhibit the metastatic ability of HCC cells in vivo.

### ROS production is responsible for the promoting effect of SHMT1 knockdown on cell motility, EMT and MMP2 production

**Fig. 5:**
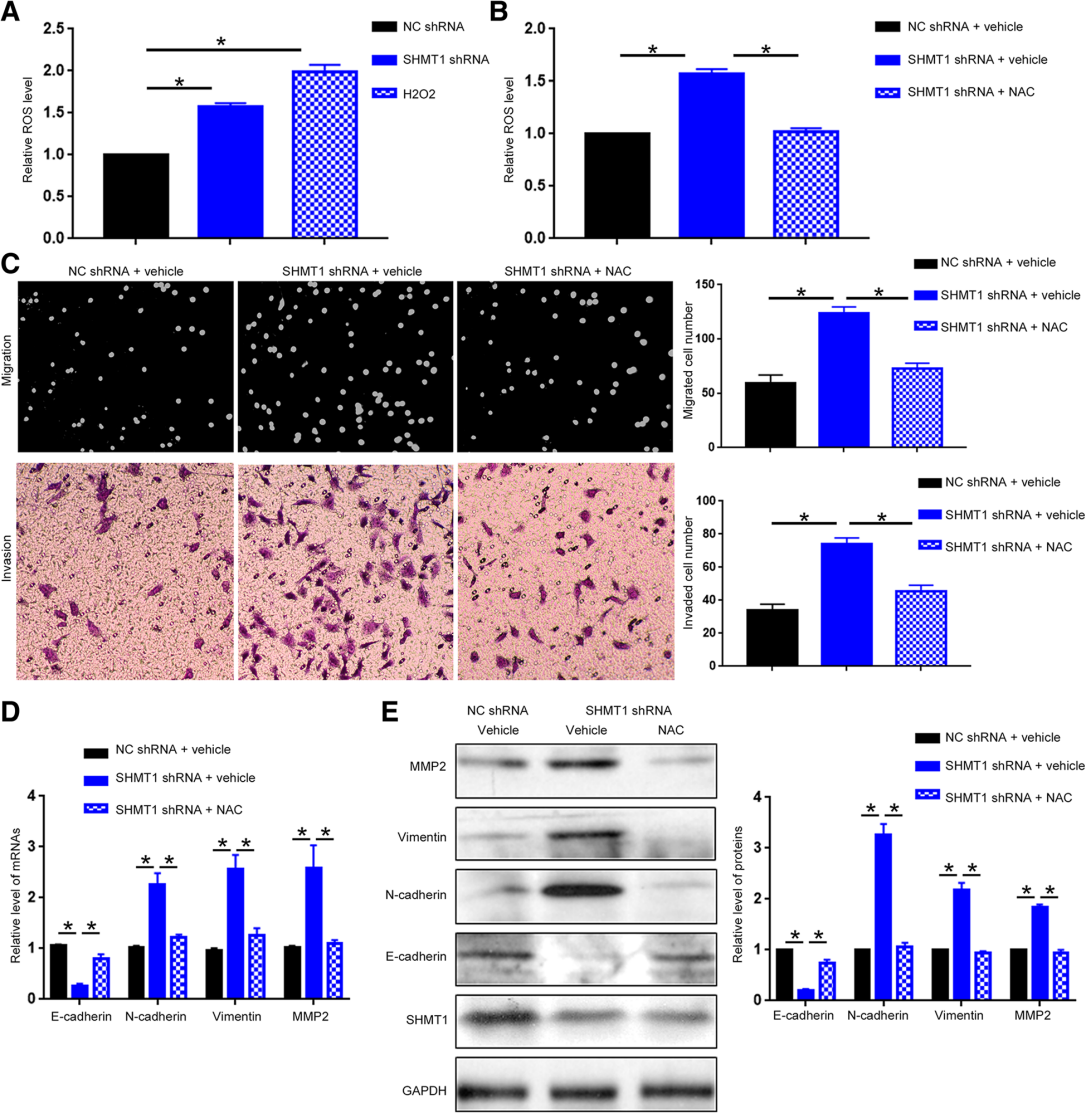
Increased ROS production is responsible for the functional influence of SHMT1 knockdown on Hep3B cells. (**a**) DCFH-DA fluorescence assay was used to investigate the effect of SHMT1 knockdown on ROS production. H_2_O_2_ treatment was used as positive control of cellular ROS production. (**b**) DCFH-DA fluorescence assay was used to examine whether NAC treatment abrogated the increase of ROS production induced by SHMT1 knockdown. (**c**) Boyden-chamber and transwell assay were performed to investigate whether NAC treatment abrogated the increase of cell migration and invasion induced by SHMT1 knockdown. (**d**) and (**e**) qRT-PCR and western blot were employed to investigate whether NAC treatment abrogated the promoting effect of SHMT1 knockdown on EMT. *P < 0.05

Previous studies showed that SHMT1 is a critical enzyme mediating the one-carbon metabolism [7, 29], which is implicated in nucleotide synthesis, DNA methylation and NADH/NADPH production in cancer cells [8, 30]. Since ROS production is closely associated with cellular NADH/NADPH level and is found to regulate the metastasis of cancer cells [31, 32], we explored whether SHMT1 affected cellular ROS production. DCFH-DA fluorescence assay was performed to evaluate the ROS level in HCC cells. As shown in Fig. 5A, SHMT1 knockdown resulted in increased DCFH-DA fluorescence, indicating increased ROS production in Hep3B cells (*P* < 0.05). To further investigate whether increased ROS production is responsible for the enhanced metastatic ability of Hep3B cells induced by SHMT1 knockdown, we treated Hep3B cell with ROS inhibitor N-acetylcysteine (NAC). NAC treatment effectively abrogated the increase of ROS production induced by SHMT1 knockdown (Fig. 5B, *P* < 0.05). Consequently, NAC treatment reversed the increase of migration and invasion induced by SHMT1 knockdown in Hep3B cells (Fig. 5C, *P* < 0.05). qRT-PCR and western blot showed that the decrease of E-cadherin and the increase of N-cadherin, vimentin and MMP2 induced by SHMT1 knockdown was abrogated by NAC treatment (Fig. 5D and E, *P* < 0.05). Therefore, these data indicate that increased ROS production was responsible for the enhanced cell motility, EMT and MMP2 expression induced by SHMT1 knockdown.

### SHMT1 regulates NOX1 expression in HCC

**Fig. 6:**
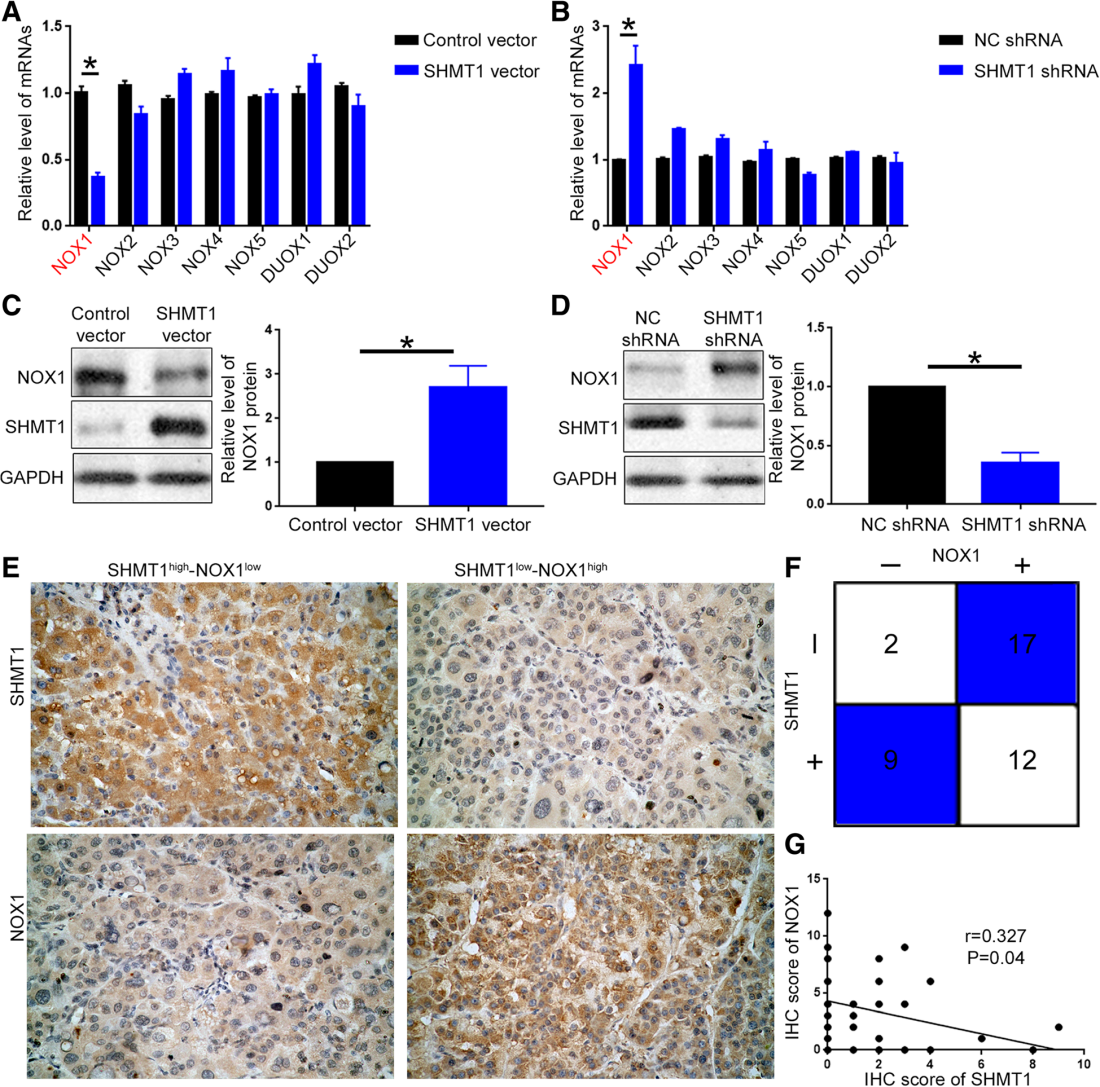
SHMT1 suppresses NOX1 expression in HCC cells. (**a**) and (**b**) qRT-PCR was performed to examine the effect of SHMT1 overexpression and knockdown on the mRNA levels of NOX family. (**c**) Western blot was performed to examine the effect of SHMT1 overexpression and knockdown on the protein level of NOX1 in HCCLM3 and Hep3B cells. (**e**) IHC staining was performed to investigate the correlation between the protein levels of SHMT1 and NOX1 in HCC tissues. (**f**) Positive rates of NOX1 staining was compared between HCC tissues with positive SHMT1 staining and those with negative SHMT1 staining using Chi-square analysis. (**g**) Correlation between the IHC scores of SHMT1 protein and NOX1 protein was evaluated using Spearman rank analysis. *P < 0.05

Mitochondria and NADPH oxidases of the Nox family including NOX1, NOX2, NOX3, NOX4, NOX5, DUOX1 and DUOX2 [33], are two important sources of ROS production in cancer cells. Therefore, we first speculated whether SHMT1 affected cellular ROS by influencing mitochondrial ROS production. MitoSox immunofluorescence staining showed that SHMT1 overexpression or knockdown did not have obvious effect on mitochondrial ROS production (Additional file 6: Figure S5A and 5B). Measurement of mitochondrial membrane potential (MMP) demonstrated that SHMT1 alteration did not have significant influence on MMP (Additional file 6: Figure S5C and 5D). This indicate mitochondria is not the key player in SHMT1 regulated ROS production in HCC cells. Thus, we further explore whether SHMT1 regulated ROS production by regulating NADPH oxidase expression. qRT-PCR screening assay was performed to examine the effect of SHMT1 on the expression of NADPH oxidase. qRT-PCR for the 7 NADPH oxidases showed that SHMT1 overexpression led to decreased mRNA level of NOX1 while its knockdown resulted in increased level of NOX1 (Fig. 6A and B, *P* < 0.05). Alteration of SHMT1 expression did not have any obvious effect on the expression of other NADPH oxidases (Fig. 6A and B). These indicate that NOX1, instead of other NADPH oxidases, is potentially a downstream target of SHMT1. Western blot analysis further confirmed that overexpression of SHMT1 led to decreased protein level of NOX1 in HCCLM3 cells (Fig. 6C, *P* < 0.05). Knockdown of SHMT1 resulted in significantly increased level of NOX1 in Hep3B cells (Fig. 6D, *P* < 0.05). To further validate the regulatory effect of SHMT1 on NOX1, we performed IHC staining of NOX1 and SHMT1 in HCC tissues. As shown in Fig. 6E, HCC tissues with high SHMT1 level showed significantly decreased staining intensity of NOX1 protein compared with those with low SHMT1 level. The positive rate of NOX1 staining in HCC tissues with negative SHMT1 staining was significantly higher than that in those with positive SHMT1 staining (Fig. 6F, *P* < 0.05). Correlation analysis showed that IHC score of SHMT1 protein was negatively correlatively with that of NOX1 protein (Fig. 6G, *P* < 0.05). In all, these data demonstrate that NOX1 expression is under the regulation of SHMT1 in HCC.

### NOX1 promotes the cell motility, EMT, MMP2 and ROS production of HCC cells

**Fig. 7:**
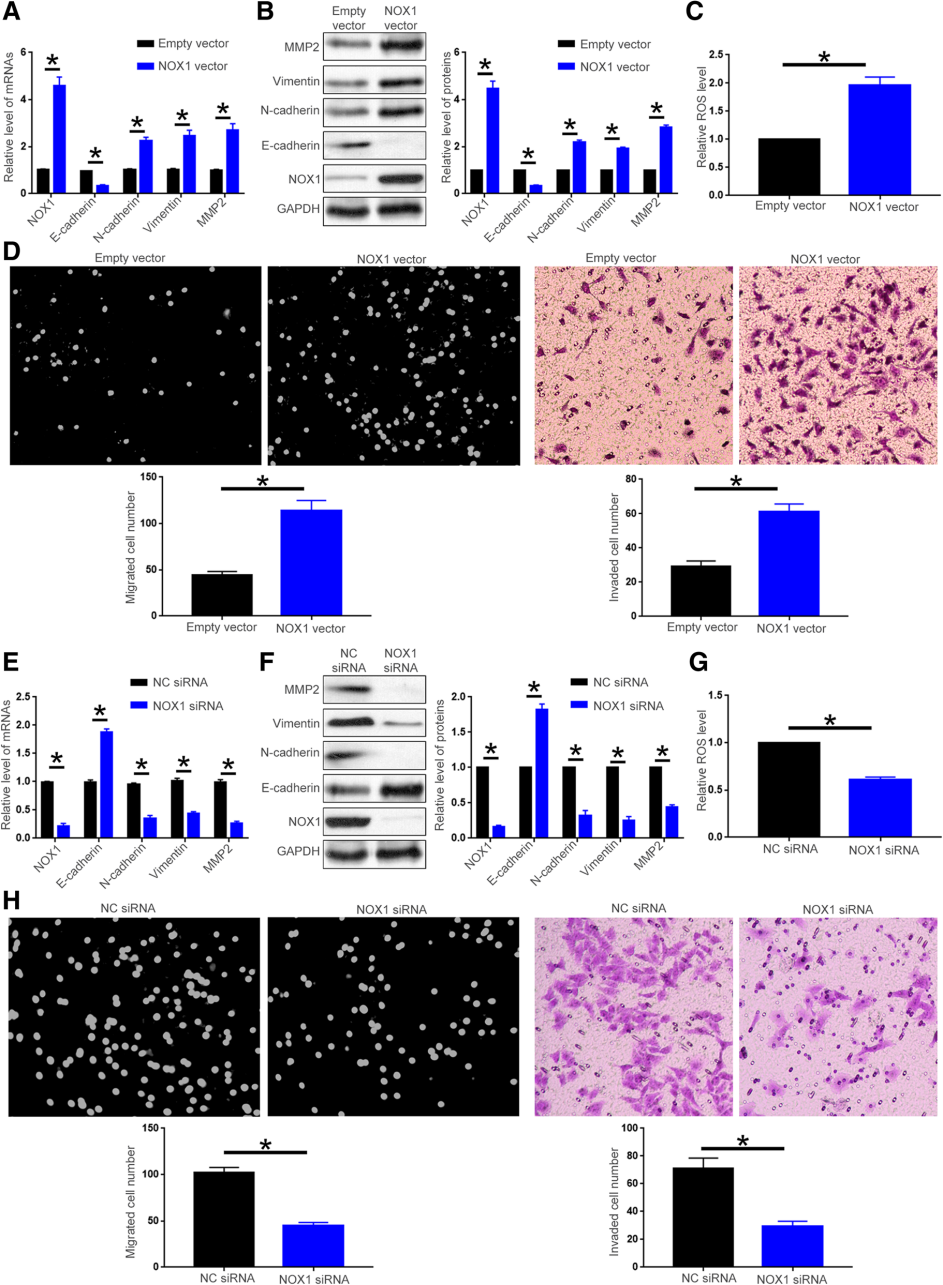
NOX1 promotes the migration, invasion, EMT and MMP2 production of HCC cells. Empty vector or NOX1 vector was transfected into Hep3B cells. (**a**) qRT-PCR and (**b**) western blot were used to evaluate the efficacy of NOX1 vector transfection, and to evaluate the effect of NOX1 overexpression on the expression level of E-cadherin, N-cadherin, Vimentin and MMP2. (**c**) DCFH-DA fluorescence assay was performed to investigate the effect of NOX1 overexpression on ROS production. (**d**) Boyden chamber and transwell assay were performed to investigate the effect of NOX1 overexpression on cell migration and invasion. Negative control siRNA or NOX1 siRNA was transfected into HCCLM3 cells. (**d**) qRT-PCR and (**e**) western blot were performed to evaluate the efficacy of NOX1 siRNA transfection, and to evaluate the effect of NOX1 knockdown on the expression level of E-cadherin, N-cadherin, Vimentin and MMP2. (**f**) DCFH-DA fluorescence assay was employed to investigate the effect of NOX1 knockdown on ROS production. (**g**) Boyden chamber and Transwell assay were performed to investigate the effect of NOX1 knockdown on cell migration and invasion. *P < 0.05

To further explore the functional influence of NOX1 on HCC cells, we transfected Hep3B cells with NOX1 vector. qRT-PCR and western blot showed that transfection of NOX1 vector significantly increased NOX1 expression in Hep3B cells (Fig. 7A and B, P < 0.05), and resulted in decreased E-cadherin level and increased level of N-cadherin, vimentin and MMP2 (Fig. 7A and B, P < 0.05). DCFH-DA fluorescence showed that forced expression of NOX1 resulted in increased ROS level (Fig. 7C, *P* < 0.05). Functionally, NOX1 overexpression led to enhanced migration and invasion of Hep3B cells (Fig. 7D, *P* < 0.05). On the other hand, NOX1 siRNA effectively reduced NOX1 level in HCCLM3 cells (Fig. 7E and F, *P* < 0.05), and inhibited EMT and MMP2 production of HCCLM3 cells (Fig. 7E and 7 F, *P* < 0.05). Subsequently NOX1 knockdown led to decreased ROS level (Fig. 7G, *P* < 0.05) and inhibited cell migration and invasion (Fig. 7H, *P* < 0.05). Taken together, these data demonstrate NOX1 can enhance cell motility, EMT, MMP2 and ROS production of HCC cells.

### NOX1 mediates the functional influence of SHMT1 on HCC cells

**Fig. 8:**
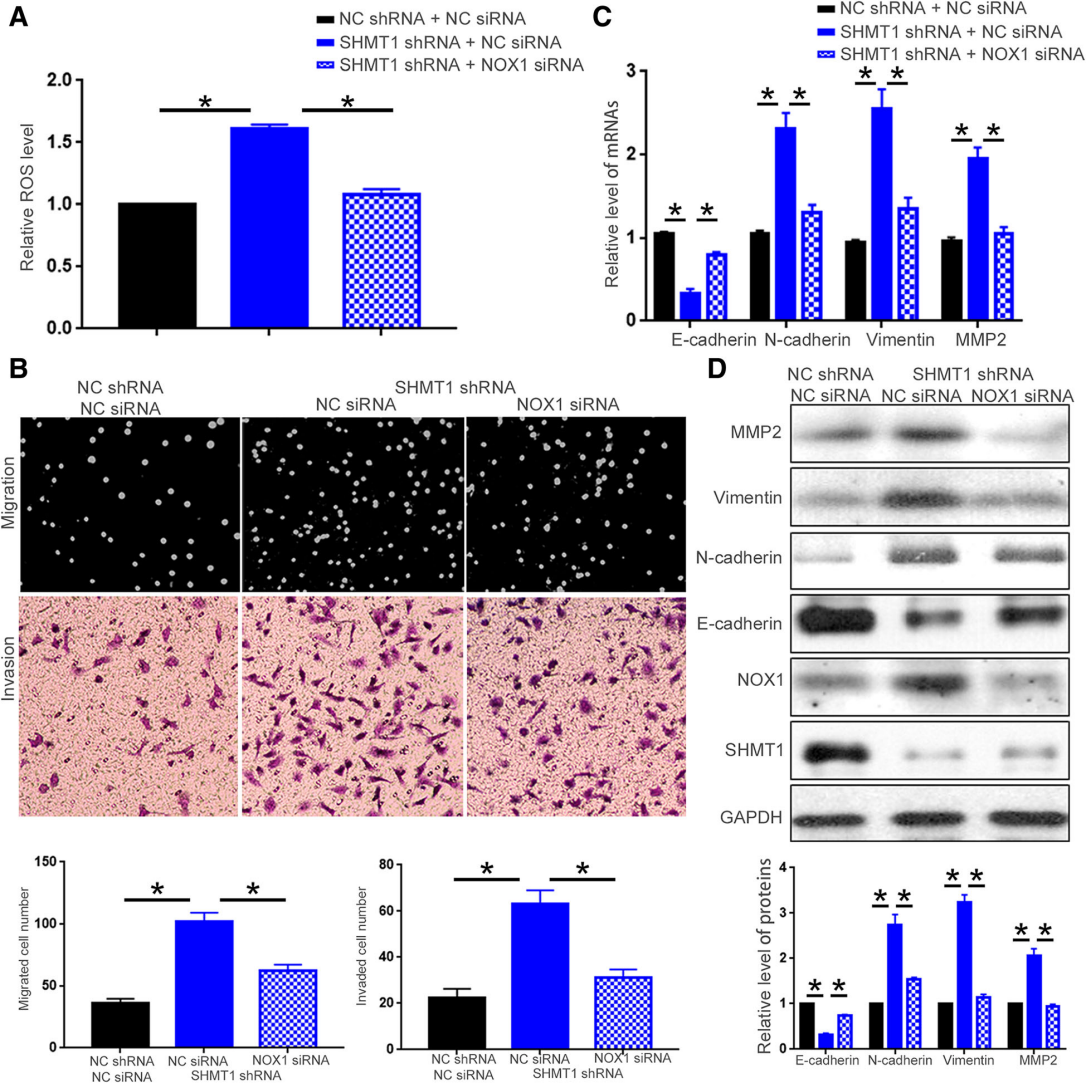
NOX1 knockdown reverses the functional influence of SHMT1 knockdown on Hep3B cells. NOX1 siRNA or negative control siRNA was transfected into Hep3B cells with or without SHMT1 knockdown. (**a**) DCFH-DA fluorescence was performed to investigate whether NOX1 knockdown blocked the promoting effects of SHMT1 knockdown on ROS production of Hep3B cells. (**b**) Boyden chamber and transwell assay were performed to evaluate whether NOX1 knockdown blocked the promoting effects of SHMT1 knockdown on the migration and invasion of Hep3B cells. (**c**) and (**d**) qRT-PCR and Western blot were performed to examine whether NOX1 knockdown blocked the promoting effects of SHMT1 knockdown on EMT and MMP2 expression of Hep3B cells. *P < 0.05

**Fig. 9:**
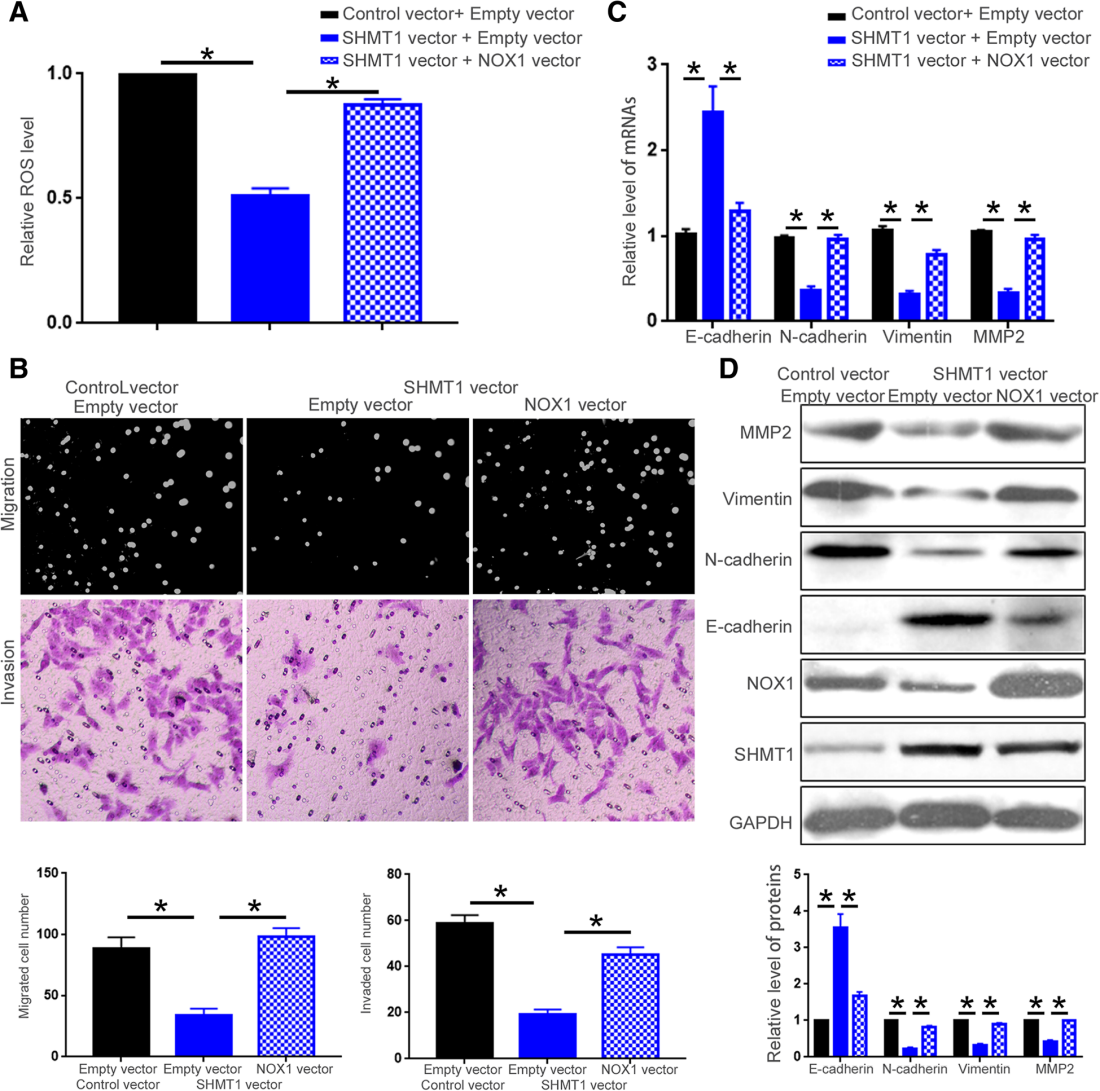
NOX1 overexpression abrogated the functional influence of SHMT1 overexpression on HCCLM3 cells. NOX1 vector or empty vector was transfected into HCCLM3 cells overexpressing SHMT1 or those in control group. (**a**) DCFH-DA fluorescence was performed to investigate whether NOX1 overexpression abrogated the decrease of ROS production induced by SHMT1 overexpression in HCCLM3 cells. (**b**) Boyden chamber and Transwell assay were performed to evaluate whether restoring NOX1 expression abrogated the decrease of cellular migration and invasion induced by SHMT1 overexpression in HCCLM3 cells. (**c**) and (**d**) qRT-PCR and Western blot were performed to examine whether restoring NOX1 expression abrogated the inhibitory effect of SHMT1 overexpression on EMT and MMP2 expression of HCCLM3 cells. *P < 0.05

To further explore whether NOX1 mediates the functional influence of SHMT1 on HCC cells, Hep3B cells with SHMT1 knockdown were transfected with NOX1 siRNA. Knockdown of NOX1 in Hep3B cells with SHMT1 knockdown suppressed the increase of ROS production resulted from SHMT1 knockdown (Fig. 8A, *P* < 0.05). NOX1 knockdown also blocked the enhancement of cell migration and invasion induced by SHMT1 knockdown (Fig. 8B, *P* < 0.05). Moreover, NOX1 knockdown partly reversed the decrease of E-cadherin and the increase of N-cadherin, vimentin and MMP2 induced by SHMT1 knockdown, as suggested by qRT-PCR and western blot (Fig. 8C and D, *P* < 0.05). On the other hand, SHMT1 overexpressing HCCLM3 cells were transfected with NOX1 vector. Forced expression of NOX1 reversed the decrease of ROS production resulted from SHMT1 overexpression (Fig. 9A, *P* < 0.05). Furthermore, restoring NOX1 expression abrogated the inhibitory effect of SHMT1 overexpression on cell migration, invasion (Fig. 9 B, *P* < 0.05) and EMT as well as MMP2 expression (Fig. 9C and D, P < 0.05). These indicate that SHMT1 regulates ROS production, cell motility, EMT and MMP2 expression of HCC cells through NOX1.

## Discussion

This study investigated the expression and biological functions of SHMT1 in HCC by analyzing the data from the publicly available databases, measuring SHMT1 level by qRT-PCR and IHC staining in the clinical specimens from our HCC patients’ cohort, and performing in vitro and in vivo experiments examining cellular metastatic ability, EMT and MMP2 expression. Our data demonstrated for the first time that SHMT1 expression was significantly decreased in HCC and inhibited the cell motility, EMT and MMP2 expression of HCC cells. More importantly, the survival data from TCGA database and our patients’ cohort showed that decreased SHMT1 expression was correlated with adverse clinicopathological characteristics and poor prognosis of HCC patients.

One-carbon metabolism, encompassing both the folate and methionine cycles and allowing cells to generate one-carbon units, is critical for the biosynthesis of anabolic precursor, methylation reaction and NADH/NADPH production [8], and has been found to play important roles in human cancers [7, 30]. SHMT1, a critical enzyme regulating one-carbon metabolism, was recently found to be a novel cancer-associated protein [9–19]. Study of triple negative breast cancer showed that positive stromal SHMT1 was correlated with poor clinical features [12]. Study of lung cancer showed that SHMT1 exerted oncogenic function by regulating cell apoptosis [9, 10]. In lacrimal gland adenoid cystic carcinoma, SHMT1 overexpression was correlated with poor prognosis [19]. SHMT1 in ovarian cancer stimulated pro-oncogenic cytokine expression to promote tumor growth and progression [11]. These data showed that SHMT1 was an oncogenic protein in these cancers. However, the expression and function of SHMT1 in HCC has never been investigated before. In this study, both the data from public databases and the results obtained from our clinical specimens consistently showed reduced expression of SHMT1 in HCC, indicating a tumor suppressive role in HCC. Functional assays further confirmed that SHMT1 exerted tumor suppressive roles in HCC by inhibiting cell metastasis, EMT and MMP2 expression. The suppressive role of SHMT1 in HCC metastasis, which was in contrary with the functions of SHMT1 in other tumors, demonstrated the cancer-type dependent function of SHMT1 in different cancers. Interestingly, a genome-wide DNA methylation and gene expression profiles in HCC demonstrated that the promoter of SHMT1 was hyper-methylated [34]. This indicates that DNA methylation is probably responsible for the reduced expression of SHMT1 in HCC.

Compared with the normal counterparts, cancer cells mostly have increased ROS production [35]. ROS levels were found to be important regulator of cancer growth, metastasis and other malignant behaviors [36]. In HCC, ROS production has been confirmed to promote the cell metastasis, EMT and MMP production of cancer cells [37, 38]. In this study, we uncovered a novel regulatory effect of SHMT1 on ROS production. SHMT1 overexpression reduced the ROS production in HCCLM3 cells while SHMT1 knockdown resulted in opposite effect in Hep3B cells. Moreover, ROS inhibitor NAC treatment blocks the promoting effect of SHMT knockdown on cell metastasis, EMT and MMP2 level. Therefore, the regulatory effect of SHMT1 on ROS production is responsible for the functional influence of SHMT1 in HCC cells. It is worth to mention here that in vascular smooth muscle cells, knockdown of SHMT1 induced oxidative stress [39]. These indicate the effect of SHMT1 on cellular ROS production is not an exclusive phenomenon in HCC cells.

NADPH oxidase, including NOX1–5, DUOX1 and DUOX2, regulates ROS production through NADPH-dependent one-electron reduction of oxygen to superoxide [40]. Recently, NOX family was found to play critical roles in human cancers [41]. Previous study of HCC showed that increased NOX1/2/5 expression was correlated with poor prognosis of HCC patients while NOX4 and DUOX1 expression predicted better overall survival of HCC patients [42]. In this study, we found that NOX1, instead of other NOX proteins, was found to under the regulation of SHMT1 in HCC. Overexpression of SHMT1 reduced NOX1 expression in HCCLM3 cells while SHMT1 knockdown led to increased NOX1 level in Hep3B cells. SHMT1 expression was negatively correlated with NOX1 expression in HCC specimens. Functional assay confirmed that NOX1 could promote the cell motility, EMT, MMP2 and ROS production of HCC cells. More importantly, knockdown of NOX1 inhibited the increase of ROS production induced by SHMT1 knockdown. Restoring NOX1 expression reversed the decrease of ROS production resulted from SHMT1 overexpression. These indicate that NOX1 mediated the regulatory effect of SHMT1 on ROS production in HCC. Functionally, altering NOX1 expression blocked the influence of SHMT1 on cell motility, EMT and MMP2 production. Taken together, this study demonstrates NOX1 not only acts as the downstream target of SHMT1 but also mediates the functional influence of SHMT1 on HCC cells. However, the molecular mechanisms by which SHMT1 regulates NOX1 expression in HCC remain unknown and are worth to be investigated in the future.

In all, this study demonstrates that SHMT1 expression is significantly decreased in HCC. Decreased SHMT1 expression is correlated with unfavorable clinical features and poor prognosis of HCC patients. SHMT1 acts as a tumor suppressor in HCC by inhibiting cell metastasis, EMT and MMP2 production. Mechanically, this study demonstrates SHMT1 inhibits ROS production in HCC cells. Moreover, SHMT1 is found to inhibit NOX1 expression in HCC cells. NOX1 mediates the regulatory effect of SHMT1 on ROS production, cell motility, EMT and MMP2 production in HCC cells. Therefore, this study reveals SHMT1 may serve as a promising biomarker and novel therapeutic target in HCC.

## Conclusions

In summary, our findings verified a novel tumor suppressor SHMT1, which was underexpressed in HCC and indicated a poor clinical outcomes of HCC patients. SHMT1 inhibited migration, invasion and EMT progression of HCC cells, and suppressed tumor metastasis of HCC in vivo. Mechanistically, SHMT1 acted as a tumor suppressor by targeting NOX1 and subsequently restrained ROS production, EMT and MMP2 expression in HCC cells. Our data suggested that SHMT1 might be a potential prognostic biomarker and therapeutic target for HCC.

## Additional files

Additional file 1: **Figure S1.** The expression and clinical significance of SHMT1 based on TCGA data. (A) TCGA data from UALCAN indicated that the expression of SHMT1 in HCC tissues was significantly lower than that in normal tissues. (B) TCGA data from “R2: Genomics Analysis and Visualization Platform” (http://r2.amc.nl) indicated that the expression of SHMT1 was reduced in HCCs with vascular invasion compared to HCCs without vascular invasion. (C) TCGA data from UALCAN revealed that advanced HCCs had a significant lower expression of SHMT1 compared to early HCCs. ^a^P < 0.05 versus Normal, ^b^P < 0.05 versus Stage I. (D) TCGA data from UALCAN revealed demonstrated that high-grade HCCs showed an obvious lower expression of SHMT1 as compared with low-grade HCCs. ^a^P < 0.05 versus Normal, ^b^P < 0.05 versus Grade 1, ^c^P < 0.05 versus Grade 2, ^d^P < 0.05 versus Grade 3. (TIF 153 kb)

Additional file 2: **Table S1.** Correlation analysis between the clinical features and SHMT1 expression in HCC (DOCX 18 kb)

Additional file 3: **Figure S2.** SHMT1 inhibits the migration, invasion, EMT and MMP2 production of Hep3B cells. Retrovirus encoding empty vector or SHMT1 vector were transduced into Hep3B cells. (A) qRT-PCR and western blot were employed to evaluate the efficacy of retrovirus transduction. (C) Boyden chamber and transwell assay were employed to investigate the effect of SHMT1 overexpression on cell migration and invasion. (TIF 1576 kb)

Additional file 4: **Figure S3.** SHMT1 did not have significant effect on the viability of HCC cells. MTT assay was performed to evaluate the effect of SHMT1 overexpression or knockdown cell viability. (A) SHMT1 overexpression in HCCLM3 cells or (B) SHMT1 knockdown in Hep3B cells did not have significant influence on cell viability. (TIF 514 kb)

Additional file 5: **Figure S4.** SHMT1 inhibits the expression of Twist1 and Snail1 in HCC cells. (A) qRT-PCR and western blot were performed to evaluate the influence of SHMT1 overexpression on the expression of Twist1, Snail1 and Zeb1. SHMT1 overexpression led to decreased expression of Twist1 and Snail1. Zeb1 expression was not significantly affected by SHMT1 overexpression. (B) qRT-PCR and western blot were performed to evaluate the influence of SHMT1 knockdown on the expression of Twist1, Snail1 and Zeb1. SHMT1 knockdown led to increased expression of Twist1 and Snail1. Zeb1 expression was not significantly affected by SHMT1 knockdown. *, *P* < 0.05. (TIF 294 kb)

Additional file 6: **Figure S5.** SHMT1 did not have significant influence on mitochondria-derived ROS and mitochondria membrane potential (MMP). MitoSox staining was performed to evaluate the effect of SHMT1 on mitochondria-derived ROS. (A) SHMT1 overexpression in HCCLM3 or (B) SHMT1 knockdown in Hep3B did not have obvious effect on mitochondria-derived ROS. (C) SHMT1 overexpression in HCCLM3 or (D) SHMT1 knockdown in Hep3B did not have obvious effect on mitochondria membrane potential. (TIF 1113 kb)

## Abbreviations

EMT: epithelial–mesenchymal transition
GEO: Gene Expression Omnibus
HCC: hepatocellular carcinoma
MMP2: matrix metalloproteinase-2
NAC: N-acetylcysteine
NOX1: NADPH oxidase 1
ROS: reactive oxygen species
SHMTs: serine hydroxymethyltransferases
