# Supplementary figures and images for: SHMT1 inhibits the metastasis of HCC by repressing NOX1-mediated ROS production
Source: J Exp Clin Cancer Res. 2019 Feb 12;38:70. doi: 10.1186/s13046-019-1067-5 (PMC6373090; doi:10.1186/s13046-019-1067-5)

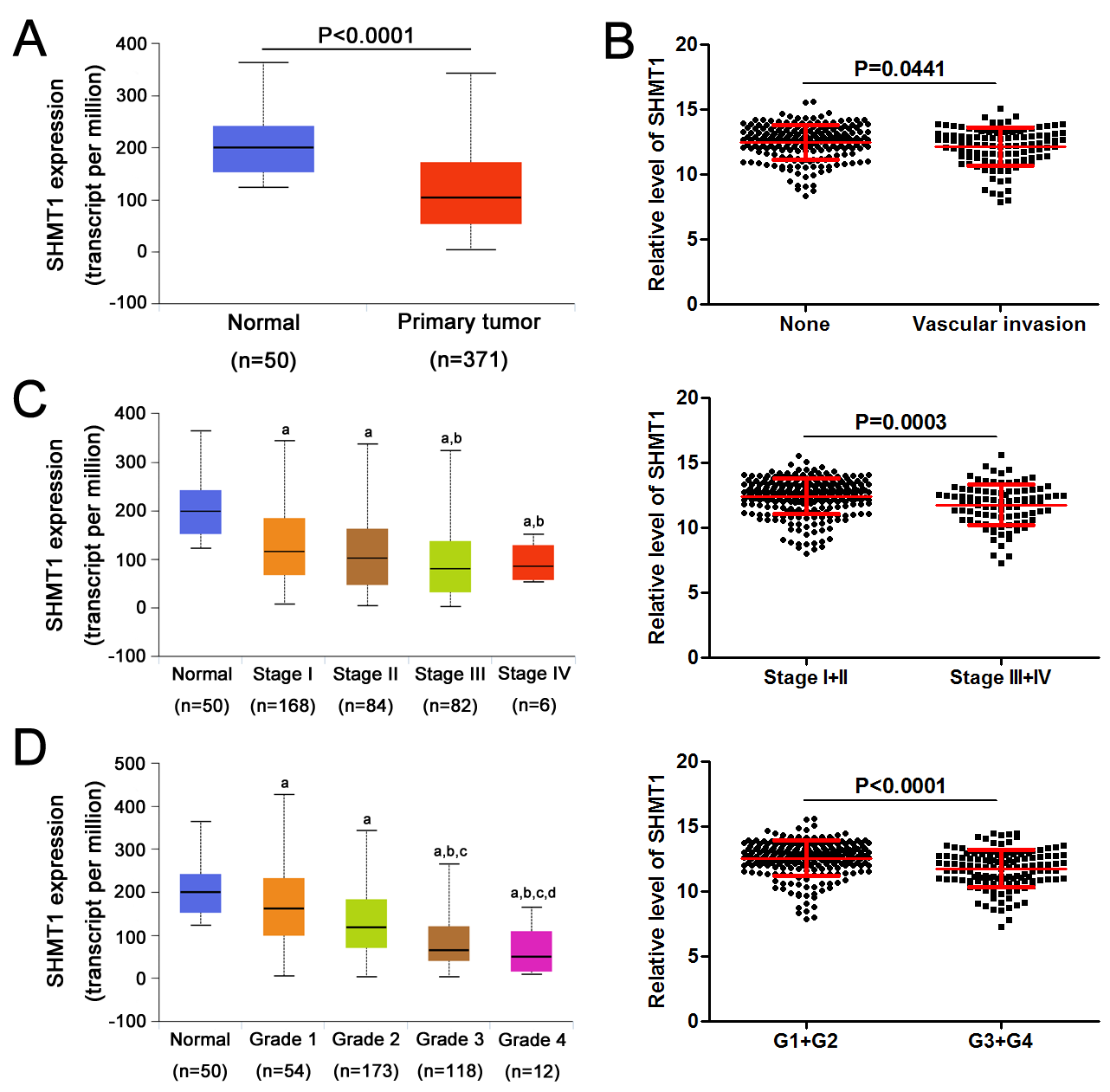

Supplement: Supplementary file 1 — Figure S1. The expression and clinical significance of SHMT1 based on TCGA data. (A) TCGA data from UALCAN indicated that the expression of SHMT1 in HCC tissues was significantly lower than that in normal tissues. (B) TCGA data from “R2: Genomics Analysis and Visualization Platform” (http://r2.amc.nl) indicated that the expression of SHMT1 was reduced in HCCs with vascular invasion compared to HCCs without vascular invasion. (C) TCGA data from UALCAN revealed that advanced HCCs had a significant lower expression of SHMT1 compared to early HCCs. aP < 0.05 versus Normal, bP < 0.05 versus Stage I. (D) TCGA data from UALCAN revealed demonstrated that high-grade HCCs showed an obvious lower expression of SHMT1 as compared with low-grade HCCs. aP < 0.05 versus Normal, bP < 0.05 versus Grade 1, cP < 0.05 versus Grade 2, dP < 0.05 versus Grade 3. (TIF 153 kb) [file 13046_2019_1067_MOESM1_ESM.tif]

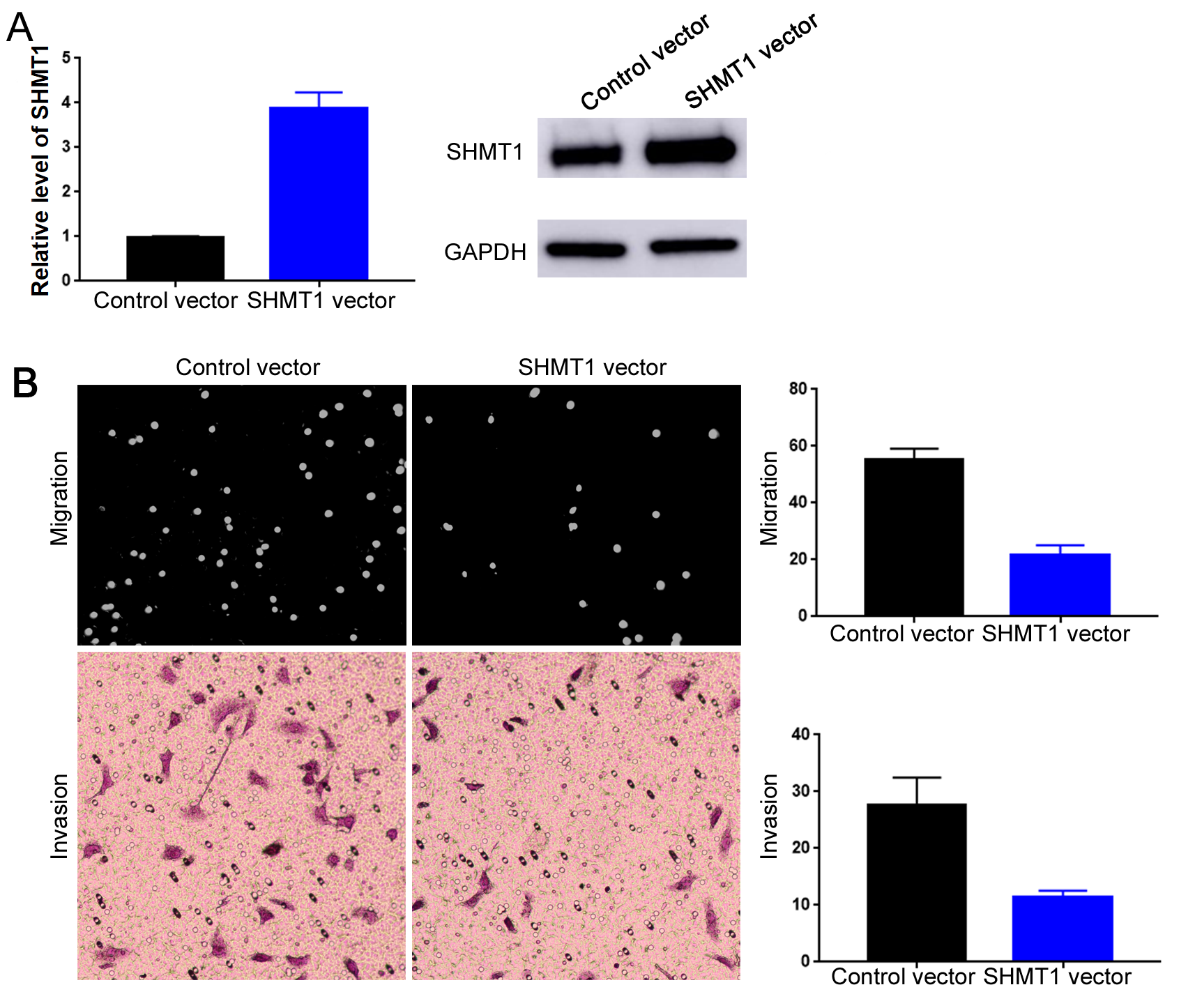

Supplement: Supplementary file 3 — Figure S2. SHMT1 inhibits the migration, invasion, EMT and MMP2 production of Hep3B cells. Retrovirus encoding empty vector or SHMT1 vector were transduced into Hep3B cells. (A) qRT-PCR and western blot were employed to evaluate the efficacy of retrovirus transduction. (C) Boyden chamber and transwell assay were employed to investigate the effect of SHMT1 overexpression on cell migration and invasion. (TIF 1576 kb) [file 13046_2019_1067_MOESM3_ESM.tif]

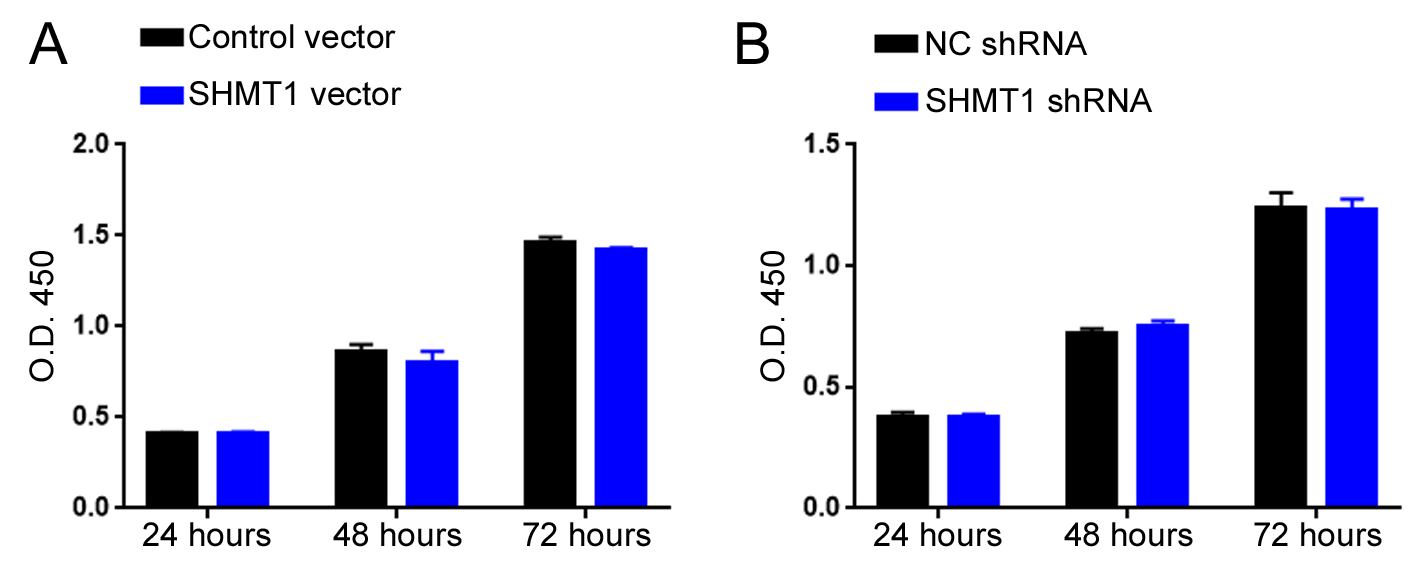

Supplement: Supplementary file 4 — Figure S3. SHMT1 did not have significant effect on the viability of HCC cells. MTT assay was performed to evaluate the effect of SHMT1 overexpression or knockdown cell viability. (A) SHMT1 overexpression in HCCLM3 cells or (B) SHMT1 knockdown in Hep3B cells did not have significant influence on cell viability. (TIF 514 kb) [file 13046_2019_1067_MOESM4_ESM.tif]

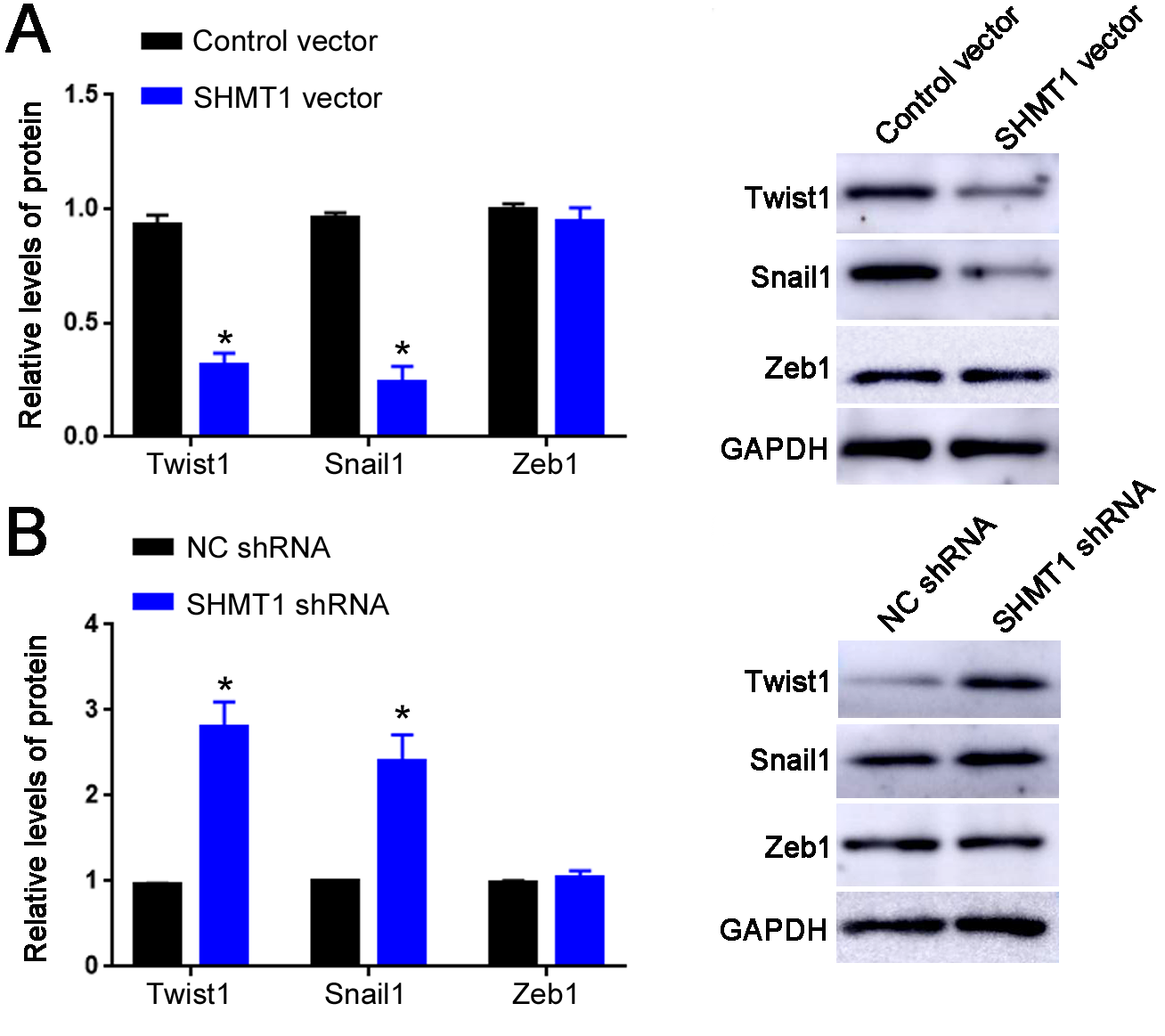

Supplement: Supplementary file 5 — Figure S4. SHMT1 inhibits the expression of Twist1 and Snail1 in HCC cells. (A) qRT-PCR and western blot were performed to evaluate the influence of SHMT1 overexpression on the expression of Twist1, Snail1 and Zeb1. SHMT1 overexpression led to decreased expression of Twist1 and Snail1. Zeb1 expression was not significantly affected by SHMT1 overexpression. (B) qRT-PCR and western blot were performed to evaluate the influence of SHMT1 knockdown on the expression of Twist1, Snail1 and Zeb1. SHMT1 knockdown led to increased expression of Twist1 and Snail1. Zeb1 expression was not significantly affected by SHMT1 knockdown. *, P < 0.05. (TIF 294 kb) [file 13046_2019_1067_MOESM5_ESM.tif]

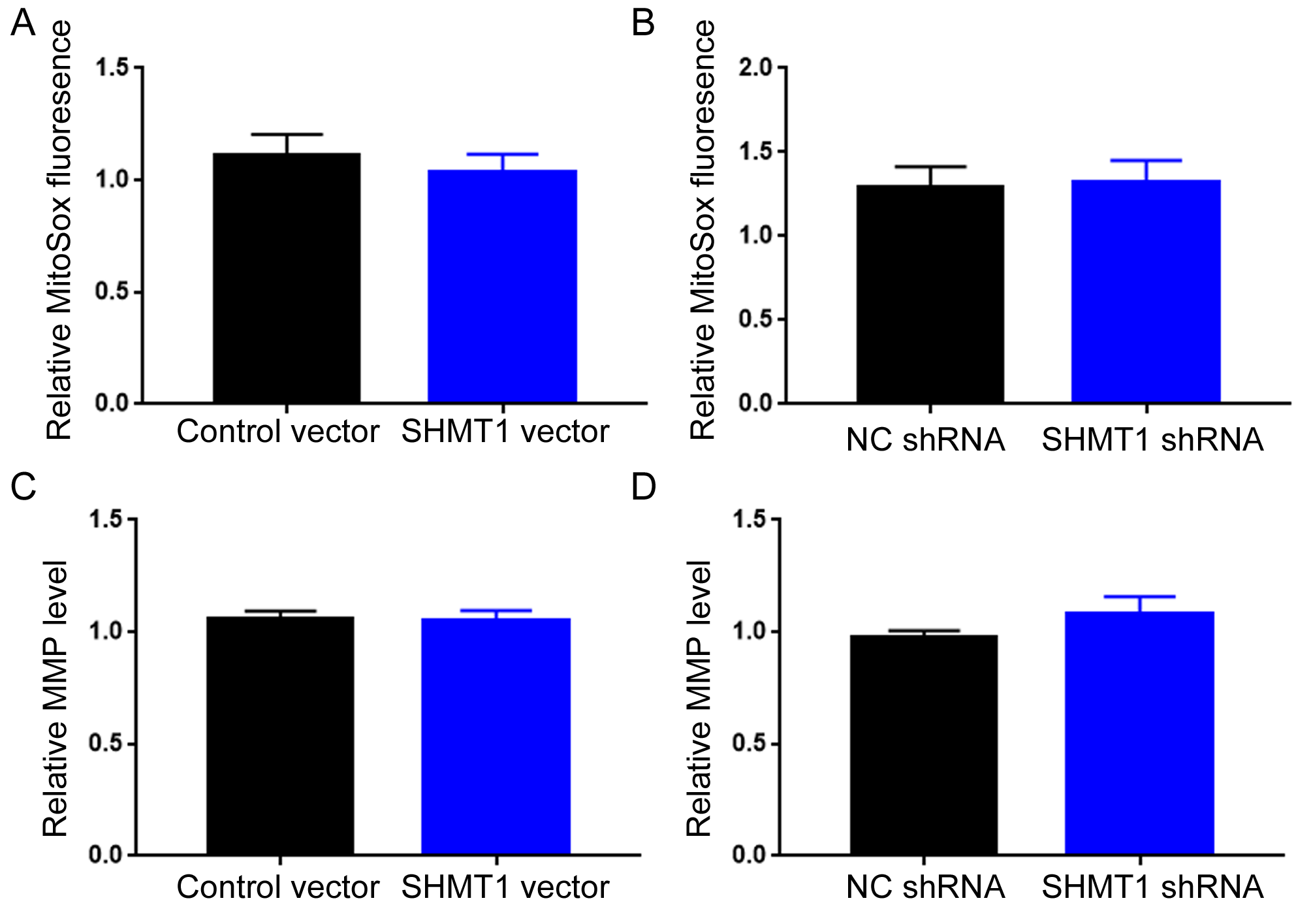

Supplement: Supplementary file 6 — Figure S5. SHMT1 did not have significant influence on mitochondria-derived ROS and mitochondria membrane potential (MMP). MitoSox staining was performed to evaluate the effect of SHMT1 on mitochondria-derived ROS. (A) SHMT1 overexpression in HCCLM3 or (B) SHMT1 knockdown in Hep3B did not have obvious effect on mitochondria-derived ROS. (C) SHMT1 overexpression in HCCLM3 or (D) SHMT1 knockdown in Hep3B did not have obvious effect on mitochondria membrane potential. (TIF 1113 kb) [file 13046_2019_1067_MOESM6_ESM.tif]
