# Supplementary material for: SHMT1 inhibits the metastasis of HCC by repressing NOX1-mediated ROS production
Source: J Exp Clin Cancer Res. 2019 Feb 12;38:70. doi: 10.1186/s13046-019-1067-5 (PMC6373090; doi:10.1186/s13046-019-1067-5)
Supplement: Supplementary file 2 — Table S1. Correlation analysis between the clinical features and SHMT1 expression in HCC (DOCX 18 kb) [file 13046_2019_1067_MOESM2_ESM.docx]

**Table S1.** Correlation analysis between the clinical features and SHMT1 expression in HCC

| **Characteristics of patients** | | **No. of patients** | **SHMT1 staining** | | ***P*** |
| --- | --- | --- | --- | --- | --- |
|  |  |  | **Negative** | **Positive** |  |
| Age (y) | <60 | 86 | 44 | 42 | 0.317 |
|  | ≥60 | 34 | 21 | 13 |  |
| Sex | Male | 103 | 58 | 45 | 0.298 |
|  | Female | 17 | 7 | 10 |  |
| HBV infection | Absent | 15 | 10 | 5 | 0.408 |
|  | Present | 105 | 55 | 50 |  |
| Cirrhosis | Absent | 23 | 13 | 10 | 0.821 |
|  | Present | 97 | 52 | 45 |  |
| AFP level | <20 | 36 | 13 | 23 | 0.016* |
|  | ≥20 | 84 | 52 | 32 |  |
| Tumor size (cm) | <5 | 44 | 19 | 25 | 0.087 |
|  | ≥5 | 76 | 46 | 30 |  |
| Vascular invasion | Absent | 56 | 39 | 17 | 0.002* |
|  | Present | 64 | 26 | 38 |  |
| Edmondson-Steiner grading | I+II | 76 | 42 | 34 | 0.850 |
|  | III+IV | 44 | 23 | 21 |  |
| TNM tumor stage | I+II | 39 | 14 | 25 | 0.006* |
|  | III+IV | 81 | 51 | 30 |  |

HCC, hepatocellular carcinoma; HBV, hepatitis B virus; AFP, alpha fetoprotein; TNM, tumor-node-metastasis. ^*^ Statistically significant.
